# Persistent Left Superior Vena Cava: Rare Location of Hemodialysis Catheter

**DOI:** 10.7759/cureus.35579

**Published:** 2023-02-28

**Authors:** Ana Piedade, Patrícia Domingues, Ana Natário, Carlos Barreto, Lúcia Parreira

**Affiliations:** 1 Nephrology, Setubal Hospital Center, Setubal, PRT

**Keywords:** hemodialysis access, vascular anomaly, central venous catheterization, catheter, left superior vena cava

## Abstract

When the left cardinal vein fails to involute during fetal life, a persistent left superior vena cava (PLSVC) develops. PLSVC is a rare vascular anomaly, and the reported incidence is 0.3-0.5% in healthy individuals. It is usually asymptomatic and does not cause hemodynamic disturbances unless associated with cardiac malformations. If the PLSVC drains adequately into the right atrium and there are no cardiac abnormalities, catheterization of this vessel, including temporary and cuffed HD catheter insertion, is deemed safe. We present the case of a 70-year-old woman with acute kidney injury (AKI), in which the necessity to place an HD central venous catheter (CVC) through the left internal jugular vein led to the discovery of a PLSVC. Once it was shown that the vessel was adequately draining into the right atrium, this catheter was changed to a cuffed tunneled HD catheter, which was successfully utilized for HD sessions for three months and removed after the recuperation of renal function without complications.

## Introduction

The central venous catheter (CVC) is a type of vascular access that is commonly used in hemodialysis patients, especially those with acute kidney injury (AKI). It is not the preferred vascular access for these patients since it can be associated with several complications, such as infection and several mechanical complications. The most frequent location for HD CVC placement is the right internal jugular vein (RIJV). The second location of choice is usually the left internal jugular vein (LIJV) [1]. Persistent left superior vena cava (PLSVC) is a rare vascular abnormality that develops when the left cardinal vein fails to involute during fetal life [2]. The reported incidence is 0.3-0.5% in healthy individuals, but it may be associated with cardiac malformations, and in patients with congenital heart disease, it may be diagnosed in 3-10% [3,4]. It is usually asymptomatic and does not cause hemodynamic disturbances unless associated with cardiac malformations [4]. PLSVC is usually detected incidentally [2].

## Case presentation

**Figure 1:**
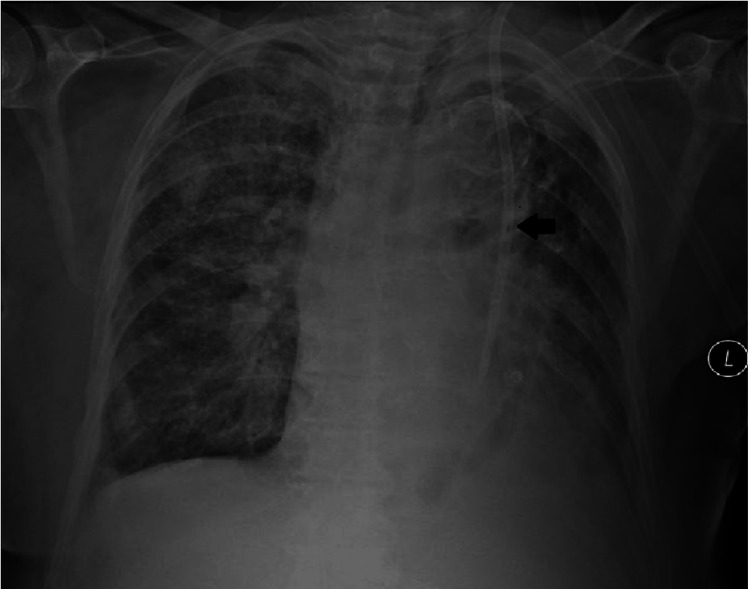
Chest radiogram with the catheter running down the left mediastinal heart border (arrow).

**Figure 2:**
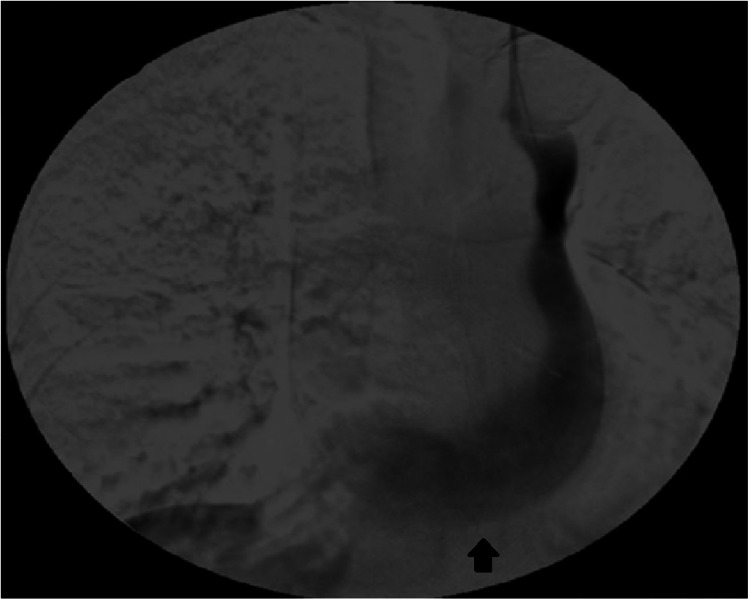
Angiography. Patency of the left superior vena cava, which drains into the right atrium via the coronary sinus (arrow).

**Figure 3:**
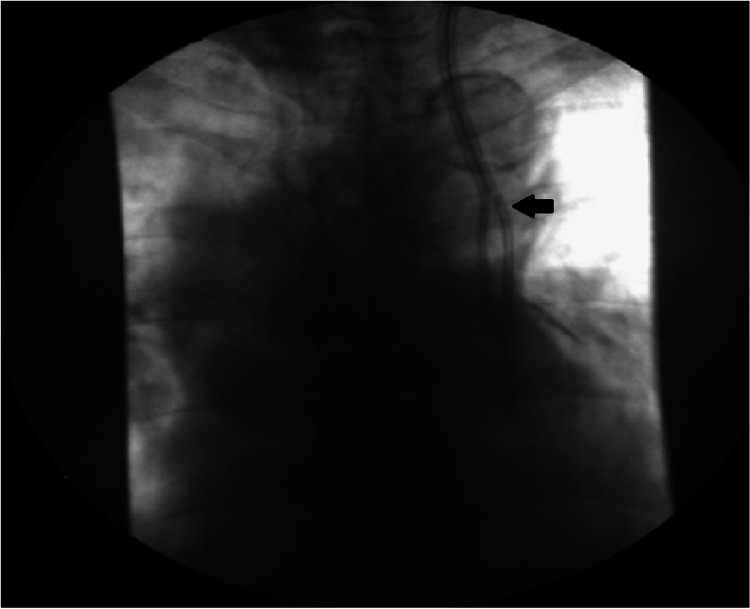
Cuffed catheter implanted through the left internal jugular vein under fluoroscopic (arrow).

We present the case of a 70-year-old woman with a medical history of arterial hypertension, obstructive sleep apnea, and breast carcinoma who had previously undergone surgery and radiotherapy. She was medicated with fluvestrant, ribociclib, and zoledronic acid due to bone metastases. Therapy was later changed to everolimus and exemestane because of liver metastases. The patient had normal renal function at the time of the last therapeutic change. One month later, the patient was admitted to the emergency department with asthenia, anorexia, and general malaise. Analytically, these values are hemoglobin 6.5 g/dL, platelets 72,000 mg/dL, creatinine 11.85 mg/dL, and urea 228 mg/dL. AKI, probably related to mTOR inhibitor toxicity, was diagnosed. Due to worsening AKI and the need for HD initiation, an uncuffed HD CVC was placed through the RIJV. Five days later, due to obstruction, there was a need to replace the RIJV HD CVC. During this procedure, accidental migration of the tip of the CVC to the right atrium occurred, which was removed with an endovascular procedure, leaving a suture at the site of the approach of the vein and making the use of RIJV unfeasible. The patient maintained the need for HD, so an uncuffed CVC was placed through the LIJV without issues and with good blood flow. The CVC was visible along the left mediastinal heart boundary on the post-insertional radiogram (Figure 1). As there was no evidence of AKI improvement and no conditions for placement of a CVC in the RIJV, an angiogram was performed, and the patency of the left superior vena cava with adequate drainage through the coronary sinus to the right atrium was verified (Figure 2). An HD-cuffed catheter was implanted through the LIJV under fluoroscopic control (Figure 3), which remained as the patient's access for HD with a good blood flow rate and without any complications. Three months later, there was an AKI resolution, and HD was suspended. The CVC was removed with no complications.

## Discussion

The left common cardinal vein, an embryologic vessel that typically involutes into the coronary sinus around the eighth week of fetal life, persists and leads to the PLSVC [2,3,5,6]. The left and right superior vena cava are present in most cases of PLSVC (>80%), but more rarely, abnormalities of the embryological development can lead to an absent right superior vena cava with left superior vena cava persistence [7]. PLSVC is the most common congenital anomaly of the thoracic vasculature while being an uncommon vascular defect [3,7]. Rarely is this reported because most CVCs are placed in the RJIV, or because the lack of knowledge of its existence can lead clinicians to remove the CVC after seeing the control radiography, thinking that it is a false path [7]. In general, it is asymptomatic, clinically undetectable, and often hemodynamically insignificant, unless associated with congenital cardiac malformations such as an atrioventricular septal defect, a ventricular septal defect, or an atrial septal defect [2,3]. The diagnosis is usually incidental, for example, during catheterizing procedures like HD CVC placement [1]. PLSVC typically does not cause symptoms, although it can make central venous catheterization difficult [3,8]. Chest radiography usually aids in the diagnosis by showing a left-sided paramediastinal course of the CVC [8,9]. If the PLSVC drains adequately to the right atrium and there are no cardiac abnormalities, catheterization of this vessel, including the implantation of temporary and cuffed HD catheters, is deemed safe [3,8,9]. Echocardiography, computed tomography, or angiography can be used to confirm that the PLSVC drains properly into the right atrium [3,10].

In our case, the impossibility of catheterizing the RIJV led to the accidental diagnosis of PLSVC. There was no evidence of cardiac malformations. By performing an angiography, it was possible to confirm correct drainage into the right atrium and catheterize the vessel. A cuffed HD CVC was placed and remained for three months as vascular access for HD, with adequate blood flow and no associated complications.

## Conclusions

The placement of central venous catheters is a frequent practice, especially by nephrologists. This case report highlights the importance of awareness of the existence of PLSVC for any clinician who frequently inserts central venous catheters into the left internal jugular vein, to avoid misinterpretation and inappropriate clinical responses to routine chest radiograms taken to confirm proper placement of such catheters. In view of the existence of PLSVC, this can be considered a safe and durable alternative to the placement of a cuffed hemodialysis catheter.

## References

[1] Schummer W, Schummer C, Fröber R (2003). Persistent left superior vena cava and central venous catheter position: clinical impact illustrated by four cases. Surg Radiol Anat.

[2] Boodhun M, Mohammad N, Adnan A, Wan Ghazali WS (2018). Catheterisation of a persistent left superior vena cava. BMJ Case Rep.

[3] Parreira LF, Lucas CC, Gil CC, Barata JD (2009). Catheterization of a persistent left superior vena cava. J Vasc Access.

[4] Sarodia BD, Stoller JK (2000). Persistent left superior vena cava: case report and literature review. Respir Care.

[5] Verniquet A, Kakel R (2012). Persistent left superior vena cava: implications during central venous cannulation. Can J Anaesth.

[6] Goyal SK, Punnam SR, Verma G, Ruberg FL (2008). Persistent left superior vena cava: a case report and review of literature. Cardiovasc Ultrasound.

[7] Kute VB, Vanikar AV, Gumber MR, Shah PR, Goplani KR, Trivedi HL (2011). Hemodialysis through persistent left superior vena cava. Indian J Crit Care Med.

[8] Wasse H (2006). Persistent left superior vena cava: diagnosis and implications for the interventional nephrologist. Semin Dial.

[9] Kuppusamy TS, Balogun RA (2004). Unusual placement of a dialysis catheter: persistent left superior vena cava. Am J Kidney Dis.

[10] Stylianou K, Korsavas K, Voloudaki A, Patrianakos A, Vardaki E, Tzenakis N, Daphnis E (2007). Can a left internal jugular catheter be used in the hemodialysis of a patient with persistent left superior vena cava?. Hemodial Int.

